# Assessment of maize nitrogen uptake from PRISMA hyperspectral data through hybrid modelling

**DOI:** 10.1080/22797254.2022.2117650

**Published:** 2022-09-05

**Authors:** Marina Ranghetti, Mirco Boschetti, Luigi Ranghetti, Giulia Tagliabue, Cinzia Panigada, Marco Gianinetto, Jochem Verrelst, Gabriele Candiani

**Affiliations:** a Institute for Electromagnetic Sensing of the Environment (IREA), National Research Council of Italy, Milano, Italy; b Remote Sensing of Environmental Dynamics Laboratory, Dipartimento di Scienze dell’Ambiente e della Terra, Università degli Studi di Milano - Bicocca, Milano, Italy; c Department of Architecture, Built Environment and Construction Engineering (DABC), Milano, Italy; d Image Processing Laboratory (IPL), Parc Científic, University of Valencia, Paterna, Valencia, Spain

**Keywords:** Precision farming, radiative transfer models, machine learning regression algorithms, plant nitrogen uptake estimation

## Abstract

The spaceborne imaging spectroscopy mission *PRecursore IperSpettrale della Missione Applicativa* (PRISMA), launched on 22 March 2019 by the Italian Space Agency, opens new opportunities in many scientific domains, including precision farming and sustainable agriculture. This new Earth Observation (EO) data stream requires new-generation approaches for the estimation of important biophysical crop variables (BVs). In this framework, this study evaluated a hybrid approach, combining the radiative transfer model PROSAIL-PRO and several machine learning (ML) regression algorithms, for the retrieval of canopy chlorophyll content (CCC) and canopy nitrogen content (CNC) from synthetic PRISMA data. PRISMA-like data were simulated from two images acquired by the airborne sensor HyPlant, during a campaign performed in Grosseto (Italy) in 2018. CCC and CNC estimations, assessed from the best performing ML algorithms, were used to define two relations with plant nitrogen uptake (PNU). CNC proved to be slightly more correlated to PNU than CCC (*R*^2^ = 0.82 and *R*^2^ = 0.80, respectively). The CNC-PNU model was then applied to actual PRISMA images acquired in 2020. The results showed that the estimated PNU values are within the expected ranges, and the temporal trends are compatible with plant phenology stages.

## Introduction

Nitrogen (N), naturally present in soil, is the most important macro-nutrient for vegetation growth and productivity (Leghari et al., 2016), and for this reason, it is commonly provided in agriculture through fertilisation, in order to maximise biomass production and yield. If nitrogen deficiency can lead to crop production shortage, fertilisation excess can produce negative effects such as plant diseases or lodging, as well as environmental threats such as groundwater leaching or atmospheric pollution (greenhouse gas emission). A recent study estimated that worldwide nitrogen use efficiency in the agro-sector is about 60%, meaning that 40% of N, provided through fertilisation, is wasted in the environment, with negative economic and environmental impacts (Lassaletta et al., 2016). Indeed, spatio-temporal information about important crop variables, such as plant nitrogen uptake (PNU − kg ha^−1^), is fundamental to assess actual plant needs and to develop smart agriculture applications devoted to improve N use efficiency, guarantee sustainable crop production and reduce environmental impacts (Basso et al., 2016; Zarco-Tejada & Loudjani, 2017).

In the last decades, remote sensing was successfully exploited to map crop nitrogen at farm level, in order to identify the variability of nutritional conditions among and within fields. Most studies derived information on crop nutritional status by estimating leaf/canopy chlorophyll content (Baret et al., 2007; Delloye et al., 2018; Guerif et al., 2007). This work-flow had a certain advantage since chlorophyll spectral absorption features are strong in the visible domain and easy to detect through multispectral data as a consequence of light harvest in the photo-synthesis process (Berger, Verrelst, Féret, Wang et al., 2020). Although a significant amount of literature presents good relationships between nitrogen and chlorophyll (Hansen & Schjoerring, 2003; He et al., 2016; Tian et al., 2011), it was also reported that this linkage is dependent on species and phenological status (Berger, Verrelst, Féret, Wang et al., 2020; Homolová et al., 2013). Additionally, it is important to remind that chlorophyll contains only a fraction of total leaf (plant) nitrogen (~19%), while the main sink consists of proteins such as rubisco (~30%; Berger, Verrelst, Féret, Wang et al., 2020; Kokaly et al., 2009). This condition does not guarantee the direct linkage between chlorophyll content and total nitrogen in plant tissue; hence, retrieval schemes based only on chlorophyll estimation may not be transferable in time and space or across species. Consequently, the direct estimation of nitrogen from proteins is foreseen as the best way to exploit remote-sensing information for crop nutritional status monitored across agricultural sites and seasons (Berger, Verrelst, Féret, Wang et al., 2020).

Among the different methods presented in the literature for the retrieval of biophysical variables (BVs), data-driven approaches, such as parametric regressions based on vegetation indices calculation (Cilia et al., 2014; Clevers & Gitelson, 2013; Nutini et al., 2018; Stroppiana et al., 2009) or machine learning regression algorithm (MLRA) techniques (Verrelst et al., 2015, 2019), are the most commonly used (Liu et al., 2021). Despite the good results, the intrinsic empirical nature of these approaches bounds their use to the specific experimental conditions presented in each study, limiting the transferability to different contexts (including space, time and plant type/crop species). To overcome this issue, physically based methods were proposed for BV estimation through the inversion of radiative transfer models (RTMs). RTMs are based on equations devoted to reconstruct the vegetation reflectance spectrum, as measured by an Earth Observation sensor, considering target features such as vegetation parameters at both leaf and canopy level, background characteristics and illumination/viewing geometry conditions. The estimation of BVs from RTMs can be performed by means of iterative numerical optimization or inversion based on look-up-tables of vegetation spectra (Verrelst et al., 2019). Nonetheless, BV retrieval from RTMs remains challenging due to its expensive computational requirements. The hybrid approach, recently introduced by the scientific community for BV retrieval (Berger, Verrelst, Féret, Hank et al., 2020; Verrelst et al., 2020, 2019), represents a possible solution to this problem. This approach consists in the combination of RTM and MLRA: RTM generates a database of simulated vegetation spectra (input), related to the vegetation BVs (output), and MLRA identifies a non-linear model between these input−output pairs. Thus, hybrid methods inherit the generalisation of physically based methods as well as the flexibility and computational efficiency provided by MLRAs. A complete and exhaustive review on this topic is covered in Berger et al. (2020).

Recently, Féret et al. (2021) provided a modification of the well-known leaf-level RTM PROSPECT (Jacquemoud & Baret, 1990) by separating the contribution of proteins (C_p_) and carbon-based constituents (CBCs) on leaf reflectance, thus allowing to simulate the effect of protein content on leaf spectra. This innovation opens new perspective for the retrieval of leaf protein content as a proxy of plant nitrogen uptake directly from spectroradiometric data. To perform N retrieval from C_p_, high spectral resolution data in the full spectral range are needed: besides the mentioned chlorophyll related features in the visible and near infrared (VNIR) spectral region, the main protein absorptions are in the shortwave infrared (SWIR) domain (Berger, Verrelst, Féret, Wang et al., 2020; Kokaly et al., 2009; Z. Wang, Skidmore, Wang, Skidmore, Wang et al., 2015). Hyperspectral data are therefore the candidate data source for protein estimation (Rast et al., 2019), and hybrid approaches are the state-of-the-art solution for such retrieval scheme. This approach for canopy nitrogen content (CNC) retrieval was recently tested using spectroradiometric field data (Berger, Verrelst, Féret, Hank et al., 2020), simulated hyperspectral satellite imagery (Candiani et al., 2022) and actual hyperspectral satellite data, providing the first landscape-level map (Verrelst et al., 2021) as well as multitemporal and multi-crop analyses (Tagliabue et al., 2022). The foreseen hyper-spectral data stream from space, provided by already launched satellites, such as PRISMA (Loizzo et al., 2016, 2019) from the Italian Space Agency (ASI) or EnMap (Chabrillat et al., 2020) from the German Space Agency (GFZ-DLR), or future satellite mission planned in the upcoming years, such as CHIME (Nieke & Rast, 2019) from the European Space Agency (ESA) or SBG (Thompson et al., 2020) from the National Aeronautics and Space Administration (NASA), will open new opportunities in crop monitoring, sustainable agriculture and precision farming (Hank et al., 2019). In this framework, it is important to study and develop new efficient methods for the operational mapping of important crop traits, such as chlorophyll and nitrogen content, exploiting the already available hyperspectral satellite data (i.e. PRISMA).

Consequently, it is necessary to further test the potentiality of hybrid approach to assess canopy chlorophyll and nitrogen content, to evaluate the performance of different MLRAs and the impact of hyperspectral feature selection on BV retrieval. Moreover, it is necessary to set up operational workflows for PNU estimation and to analyse the advantage of the canopy nitrogen content retrieved from proteins with respect to traditional approaches based on chlorophyll estimation. Finally, generation and analysis of multitemporal maps are required to fully assess the capability of imaging spectroscopy to estimate PNU, as an added value for sustainable agriculture.

Therefore, this work develops a hybrid approach for the estimation of maize canopy chlorophyll content (CCC) and canopy nitrogen content (CNC) from actual PRISMA data with the final goal to (i) evaluate the retrieval performance of hybrid models for CCC and CNC, (ii) compare the relations of these BVs with PNU and (iii) demonstrate PRISMA capabilities to map PNU.

## Materials and methods

### General workflow of the study

**Figure 1:**
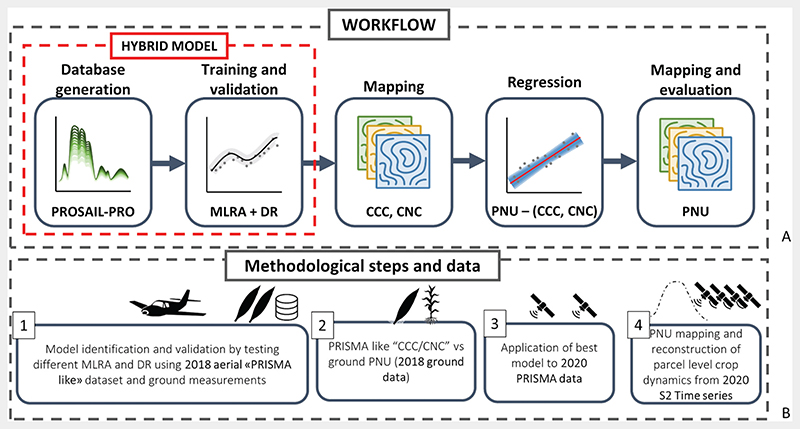
General workflow of the methodological phases followed in this study.

Figure 1 provides a synthetic representation of the general workflow of this study (top panel − A) and the method and data specifically used for each step (bottom panel − B). The first step consists in the set-up of hybrid models for PRISMA (database generation and training and validation of different MLRA and dimensionality reduction − DR combination) for the estimation of CCC and CNC. The best hybrid models for CCC and CNC from cross-validation were applied to PRISMA-like data, obtained by HyPlant-DUAL hyperspectral airborne images, to compare BV maps with ground data (Step 1). Specific regressive models between CCC and CNC and PNU were defined using 2018 ground data and PRISMA-like estimations (Step 2). Finally, the best hybrid model for BV retrieval was applied to actual PRISMA data acquired on the same study area on 2020 (Step 3). The best BV−PNU linear model was then applied to BV maps to generate PNU maps. PNU crop dynamics at parcel level was evaluated comparing ranges of parameter estimation to crop phenological status and development as derived from time series of Sentinel-2 data (Step 4). Further details will be given in the following sections.

### Study area and field measurements

**Figure 2:**
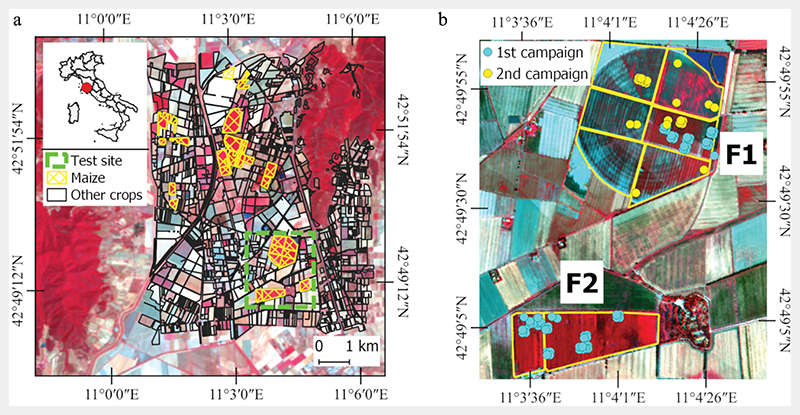
Study area and field measurements. Panel (a) shows the study area, field boundaries and maize cultivation (yellow-meshed colour) for 2020, 1 August 2020 PRISMA false colour image on the background. Panel (b) indicates the test fields showing the ESU of the first (light blue points) and second (yellow points) field campaigns, 31 July 2018 HyPlant-DUAL false colour image on the background.

The study area is located in Tuscany (42°49′47.02” N 11°04′10.27” E; elev. 2 m a.m.s.l.), Central Italy, North of Grosseto, at 20 km far from the coastline (Figure 2). The site consists of a large flat irrigated area where different crops are cultivated. Within the study area, two maize crops of approximately 76 ha (F1) and 33 ha (F2) were selected as test sites. These two fields feature different irrigation systems and different sowing dates. In particular, because of its extension, F1 was divided into subzones, each sown at different dates.

During June and July 2018, in the framework of the FLuorescence EXplorer mission of the European Space Agency (ESA-FLEX) project, two field campaigns were carried out on the two fields, in order to collect a comprehensive dataset of biochemical and biophysical parameters. Independent measurements were conducted on 87 Elementary Sampling Units (ESU) of 10 × 10 m^2^, following international protocols and guidelines, as proposed by the CEOS LPV group (Morisette et al., 2006) and the VALERI project (Weiss, 2006).

Leaf biochemical variables, such as chlorophyll content (LCC), nitrogen concentration (N%) and leaf mass per area (LMA), were measured from the last fully developed leaf from three plants collected on a subset of 31 ESUs. Laboratory extractions of LCC, N% and LMA were performed on a set of three disks with 2.2 cm diameter (total area 11.40 cm^2^) sampled from each leaf. In addition, for all the 87 ESUs, indirect measurements of leaf chlorophyll were acquired using a SPAD-502 chlorophyll meter (Konica Minolta, Japan). LCC values from laboratory extractions (71 samples) and the corresponding SPAD measurements were used to identify the SPAD−LCC relationship (*R^2^* = 0.93):

(1) $$\text{LCC}\left[\mu \text{g}\;c{\text{m}}^{-2}\right]=8.24{\text{e}}^{0.0324\cdot \text{SPAD}}$$

Leaf nitrogen content (LNC) was calculated from N% and LMA according to the following equation:

(2) $$\text{LNC}\left[{\text{mgcm}}^{-2}\right]=10\cdot {\text{N}}_{\text{mass}}\cdot \text{LMA}$$

In addition, leaf area index (LAI) was measured in all ESUs using either LAI2200 plant analyser (LI-COR Biosciences, USA) or digital hemispherical photography (Jonckheere et al., 2004; Weiss et al., 2004), depending on the plant development stage. LAI values were then used to derive CCC and CNC according to the following equations:

(3) $$\text{CCC}\left[{\text{gm}}^{-2}\right]=\frac{1}{100}\cdot \text{LCC}\cdot \text{LAI}$$

(4) $$\text{CNC}\left[{\text{gm}}^{-2}\right]=10\cdot \text{LNC}\cdot \text{LAI}$$

Finally, plant density was measured in 27 ESUs. In each of these ESUs, two plants were randomly sampled for subsequent leaf and stalk dry weight measurement. Biomass was calculated by multiplying plant density by average plant dry weight (leaf plus stalks). On the same leaf and stalk samples, nitrogen concentration was also measured in laboratory using CN elemental analyser (Flash EA 1112 NC-Soil, Thermo Fisher Scientific, Pittsburgh, PA, USA), and PNC [N%] was obtained as a weighted average relative to the weight of these specific plant organs. Plant nitrogen uptake was then derived following the equation:

(5) $$\text{PNU}\left[{\text{kgha}}^{-1}\right]=100\cdot \text{Biomass}\cdot \text{PNC}$$

More details of the field and laboratory measurement protocols can be found in Candiani et al. (2022). Table 1 provides a summary of the available ground measurements.

### EO dataset

The main EO dataset is represented by PRISMA imagery. PRISMA is a medium-resolution hyperspectral imaging mission of ASI launched in 2019. The PRISMA project is conceived as a pre-operational and technology demonstrator mission, focused on the development and delivery of hyperspectral products and the qualification of the hyperspectral payload in space. The mission provides hyperspectral images at 231 bands, with a swath of 30 km and a GSD of 30 m. The spectral resolution is finer than 12 nm all over the sensor spectral range (400−2500 nm). The satellite includes a panchromatic sensor providing images in the visible spectral range, with a GSD of 5 m.

Since PRISMA was not available during the field campaign carried out in 2018, PRISMA-like data were simulated from two hyperspectral images acquired by the airborne sensor HyPlant-DUAL at the same time of the ground measurements. These images feature 480 bands with a spectral resolution (full width half maximum [FWHM]) of 3−10 nm in the spectral range 380−2510 nm and a GSD value of 1 m and 4.5 m for the scenes acquired on 7 and 30 July, respectively. The atmospheric correction was performed through an empirical line method using spectral signatures of artificial targets, vegetation, water and soil, acquired during the field campaigns (Siegmann et al., 2019). To simulate PRISMA-like data, HyPlant-Dual dataset was spectrally resampled to PRISMA wavelengths using Gaussian spectral response functions with band centres and FWHM values computed from actual PRISMA data.

The 2020 dataset includes two L2D PRISMA images acquired on 21 June and 1 August. In order to obtain smooth spectra, PRISMA images were pre-processed using RStudio® following the procedure described in Tagliabue et al. (2022) and Verrelst et al. (2021). This procedure included several steps: (i) random spikes at specific wavelengths were removed using findpeaks function included in the “pracma” package (Borchers, 2013), (ii) noisy wavelengths were excluded comparing PRISMA reflectances to ground spectra (i.e. vegetation, asphalt, and crop residues) collected with a field spectroradiometer (SR-4500; Spectral Evolution, USA); and (iii) a spline smoothing interpolation was then applied using the SplineSmoothGapfilling function implemented in the “FieldSpectroscopyCC” package (Julitta et al., 2016).

Moreover, for both simulated (Berger et al., 2018) and actual (Berger, Verrelst, Féret, Wang et al., 2020) PRISMA data, water absorption regions were excluded as well as other bands in the blue region based on considerations in Upreti et al. (2019) and Weiss, Baret et al. (2020). The final set included 150 bands in the spectral ranges 456.2−759.84 nm, 780.66− 918.92 nm, 969.3−1109.71 nm, 1163.45−1338.91 nm, 1501.74−1784.39 nm and 2019−2320.59 nm.

In addition to the PRISMA dataset, 31 Sentinel-2 (S2) level 2A images acquired from March to November 2020 were downloaded and pre-processed by means of “sen2r” R package (L. Ranghetti et al., 2021; Team, 2021) with the goal to reconstruct temporal crop dynamics to evaluate 2020 PRISMA PNU maps. The “sen2r” package was used to automatically download all the S2 images available on the study area between March and November 2020 and process them in order to resample at 10 m all the bands and to extract quality metadata (Scene Classification Map − SCL). Table 2 shows all the EO datasets described before and used in this study.

### Hybrid model set-up

The hybrid approach involves two steps: (i) the generation of a reflectance spectra database and (ii) the training of MLRAs. The RTM is used to generate a database of thousands of crop reflectance spectra according to a wide range of crop leaf and canopy parameters as well as background reflectance, viewing and illumination geometry. The advantage of using RTMs is the possibility to simulate different conditions which can be found in crop fields, hence widening the range of situations found in limited ground measurements. This database together with the associated BVs of interest is then used as input to train MLRAs. Compared to a traditional ML data-driven approach, the combination of physically based RTMs with the flexibility and computational efficiency of MLRAs should guarantee the identification of generic and robust models, hence their exportability in different contexts, enabling the operational monitoring of BVs. Table 3 shows the advantages of hybrid approach and some critical aspects to be considered when setting and using the model for crop trait retrieval and analysing the results.

### Database generation phase

The RTM used for the database generation was the PROSAIL, one of the most exploited RTMs (Jacquemoud et al., 2009) which includes the leaf model PROSPECT (Jacquemoud & Baret, 1990) and the canopy model SAIL (Verhoef, 1984). In particular, an ad-hoc MATLAB® script was developed for this study to combine the latest version of the leaf model PROSPECT (PROSPECT-PRO, Féret et al., 2021) with the canopy model 4SAIL (Verhoef, 1984; Verhoef et al., 2007).

In order to simulate leaf reflectance, the PROSPECT model requires several input parameters, describing the leaf structure (N) as well as the content of chlorophyll (LCC), carotenoid (C_cx_), anthocyanin (C_anth_), brown pigment (C_b_p) and water (Cw). Moreover, PROSPECT-PRO introduced two additional variables to differentiate the absorption behaviour of protein content (C_p_) and carbon-based constituents (CBCs), which includes cellulose, lignin, hemicellulose, starch and sugar. Leaf reflectance obtained from PROSPECT-PRO can be passed to 4SAIL to simulate the canopy reflectance in a simple way, requiring only few parameters, such as leaf area index (LAI) and average leaf angle (ALA). Additionally, the model requires information on illumination conditions and viewing geometry, such as Solar Zenith Angle (SZA), Observer Zenith Angle (OZA), relative Azimuth Angle (rAA) and the hotspot effect (Hot parameter), computed as the ratio of leaf size over canopy height (Kuusk, 1991) and the reflectance of the background below vegetation canopy (BG).

The simulations from PROSAIL-PRO were used to create a training database of 2000 maize reflectance spectra, based on different combinations of input variables, randomly sampled from either normal or uniform probability density functions, defining the crop (leaf and canopy) parameters, the soil type and the sun-sensor geometry. According to preliminary studies (M. Ranghetti et al., 2021), the size of 2000 samples was chosen as the best trade-off between accuracy and computation cost. Table 4 shows the input parameters with their symbols, units of measurement, probability distribution functions and ranges of the sampled data. The ranges of the input parameters were set according to the available field measurements or literature values (Berger et al., 2018) when field measurements were not available.

From each set of input variables, the canopy reflectance was simulated at the spectral resolution of 1 nm, ranging from 400 to 2500 nm. These spectra were then resampled at PRISMA spectral configuration (150 bands) according to the procedure described in Section “EO Dataset”. Starting from the input RTM variables, CCC and CNC were computed according to Equations (3) and (4). The database with 2000 reflectance spectra and the corresponding CCC and CNC values was used as input in the following MLRA training phase.

### MLRA training phase

During the training phase of CCC and CNC models, different combinations of MLRAs and dimensionality reduction (DR) methods were tested. The ML algorithms used in this study include Gaussian Process Regression (GPR), Artificial Neural Networks (NN), Partial Least Square Regression (PLSR), Random Forests (RF) and Support Vector Regression (SVR). Table 5 gives a brief overview of the adopted ML algorithms.

Full spectral signature has been previously adopted in retrieval approaches based on look-up table inversion (Wang et al., 2018; Zheng et al., 2018; Zhou et al., 2020) or numerical optimization strategies (Wang, Skidmore, Darvishzadeh et al., 2015; Wang, Skidmore, Wang et al., 2015). However, despite the high information content provided by hyperspectral sensors like PRISMA, thanks to hundreds of narrow bands, such datacube is affected by the so-called curse of dimensionality (Hughes phenomenon; Hughes, 1968) and is characterised by spectral redundancy and noise, which may lead to suboptimal regression performances (Verrelst et al., 2019). For such reason, DR strategies are proposed in the literature to mitigate this problem and improve model efficiency (Bruce et al., 2002; Mojaradi et al., 2009; Rivera-Caicedo et al., 2017). Different approaches are available based on band selection, either expert based (Wang et al., 2015) or automatic (Verrelst, Rivera et al., 2016) and feature transformation (Candiani et al., 2022; Pepe et al., 2020). A recent paper by Pascual-Venteo et al. (2022) performed an analysis of different DR methods, comparing band selected by statistical ranking against principal component analysis (PCA) in a hybrid retrieval approach. Results show that PCA with 20 components, explaining about 99.95% cumulative variance of the full spectral data, slightly outperformed band ranking in the retrieval of all the considered variables. On the basis of these results, PCA was considered as the most promising DR strategy to be exploited in the hybrid approach. This technique transforms a large set of variables (hyperspectral bands) into a new one where the first components contain most of the information of the original set. Reducing the number of variables in a dataset naturally comes at the expense of accuracy, but smaller datasets are easier to explore and visualise and make ML computation faster. Therefore, different numbers of components (PCA-5, PCA-10, PCA-15 and PCA-20) were tested as input to ML to assess their impact on model performance.

In addition, the database is simulated without considering different environmental and instrumental uncertainties, such as sensor or instrument noise, RTM assumption, radiometric calibration, atmospheric and geometric correction (Baret et al., 2007); therefore, the addition of noise before MLRA training phase helps to generalise the pure RTM model outputs. As such, a Gaussian white noise of 5% was added to the parameters and canopy reflectances.

At the end of this process, the performance of all 40 model combinations (5 MLRAs × 4 DRs × 2 BVs) was assessed through a *k*-fold cross-validation technique, with *k* = 10. In particular, the analysed metrics are the following: Mean Absolute Error (MAE), Root Mean Squared Error (RMSE), RMSE relative to mean observed values (rRMSE), RMSE normalised with respect to observed range values (nRMSE) and the Coefficient of Determination (*R*^2^).

### BV validation and their relationship with PNU

The best performing trained models for CCC and CNC were then applied to the 2018 datasets reported in Table 2. The independent validation of estimated CCC and CNC data was performed against ground data measured during the ESA-FLEX campaign carried out in 2018 according the same error metrics computed in cross-validation phase. Among these error metrics, MAE was chosen as an indicator to assess the best-performing algorithm for CCC and CNC, respectively. All the steps involving model training, validation and generation of maps were performed through ARTMO (Verrelst et al., 2021). Orthogonal regression analysis (reduced major axis [RMA]) was adopted to analyse performance and identify a predictive relation between PRISMA-derived CCC or CNC and field measurements of PNU. The MATLAB® gmregress function was used for this analysis (Trujillo-Ortiz & Hernandez-Walls, 2010). RMA regression is specifically formulated to handle errors in both the x (independent − BVs) and y (dependent − PNU) variables. The method was proposed in the literature for estimation of biophysical variables by taking into account uncertainty in both ground and remote measures (Berterretche et al., 2005; Cohen et al., 2003).

### PNU map demonstration

In order to demonstrate hybrid approach and PRISMA capability to map PNU, the best hybrid model for BV estimation, identified in the previous step, was applied to the actual 2020 PRISMA dataset. The BV−PNU relation was then used to convert the BV maps into PNU maps, over the agricultural study area.

In order to investigate the temporal behaviour of the considered crops and to extract remarkable crop dates, Sentinel-2 dataset acquired in 2020 over the same area of interest was also exploited to extract and analyse time series of the Modified Soil Adjusted Vegetation Index 2 (MSAVI_2_, hereinafter referred as MSAVI; Qi et al., 1994).

In particular, the R package “sen2rts” (L. Ranghetti et al., 2021) was exploited to extract MSAVI time series over the boundaries of 39 maize fields according to official regional information (http://dati.toscana.it/dataset/artea-piani-colturali-grafici-annualita-2020, accessed 5 November 2021). Raw values were smoothed using a Savitzky-Golay lowpass filter, using the SCL layer to weight them based on their associated quality. Maize cropping cycles were identified and interpolated with a double logistic function, from which the following relevant dates were computed:

- Start of season (SOS), as the date in which the first derivative of the growing curve reached the 10% of its maximum value;
- Peak of production (POP), as the date of occurrence of the maximum interpolated MSAVI value;
- End of season (EOS), as the date in which the descending curve reached the 50% of its relative value.

These phenological dates were used to interpret PNU values, estimated at the two PRISMA acquisition dates, according to crop development. Besides the assessment of absolute estimation values (ranges in relation to expected values) and interpretation of consistency of produced maps in terms of identified spatial patterns (within field variability), phenological analysis was devoted to assess reliability of estimated PNU temporal variation.

## Results

### Hybrid model performance for CCC and CNC

Generally, good results were obtained in cross-validation for all algorithms with their tested dimensionality reduction (PCA) with *R*^2^ values ranges between 0.90−0.97 and 0.56−0.68 and MAE ranges between 0.05−0.11 g m^−2^ and 0.80−0.91 g m^−2^ for CCC and CNC, respectively. GPR resulted in the most suitable algorithm, followed by NN, RF and finally SVR and PLSR. Moreover, for all the tested algorithms, except for PLSR, PCA-15 and PCA-20 led to slightly superior results than using PCA with less components (PCA-5 and PCA-10). The performances of the hybrid ML algorithms in cross-validation configuration are resumed in Tables 6 and 7. According to cross-validation metrics (MAE and *R*^2^), the best algorithm configuration was represented by GPR (as ML) with PCA20 (as DR), for both BVs.

### BV validation and PNU relation

**Figure 3:**
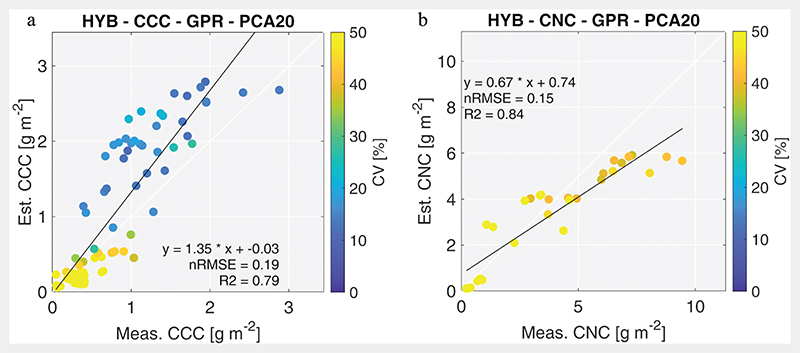
CCC (a) and CNC (b) validation scatterplots. CV represents the coefficient of variation.

These models were used to map CNC and CCC from PRISMA-like images. The estimations of CCC and CNC were validated using, respectively, 87 and 31 field measurements, available from the Grosseto 2018 dataset. Good results were obtained for CCC and CNC with *R*^2^ around 0.79 and 0.84 and nRMSE around 19% and 15%, respectively. Figure 3 shows the scatterplots between measured and estimated BVs. In general, CCC resulted in somewhat overestimated, whereas CNC retrieval was slightly underestimated. Moreover, the scatterplot highlights higher uncertainties in the CNC retrieval, featuring higher coefficient of variation values than those associated to CCC retrieval.

**Figure 4:**
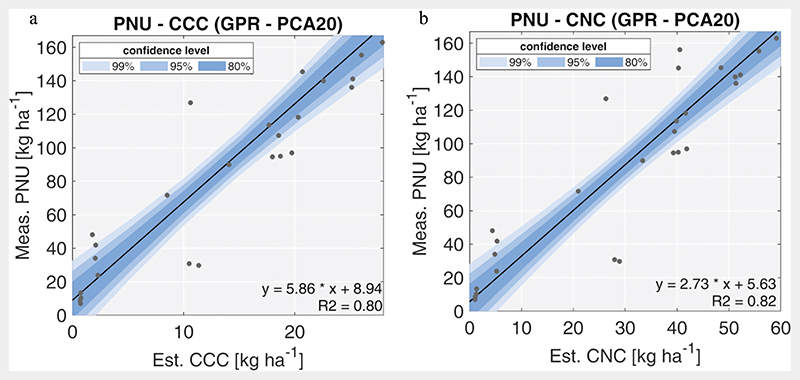
PNU correlation with CCC (a) and CNC (b). Black line represents the fit between estimated CCC and measured PNU values; blue areas identify the confidence levels of 80%, 95% and 99%.

Both BV estimations resulted in strongly correlated to ground measurements of PNU (Figure 4), with CNC showing a slightly better correlation with respect to CCC (***R***^2^ = 0.82 and ***R***^2^ = 0.80, respectively). Moreover, remote-sensed CNC is a direct estimation of a fraction of PNU, representing the organ-specific uptake from the leaves. In this view, the underestimation reported in the plot is an expected behaviour (Bender et al., 2013; Berger, Verrelst, Féret, Hank et al., 2020). Indeed, this characteristic of CNC, together with the slightly better correlation with PNU, supports this BV being potentially more important than traditional CCC to assess maize nutritional status.

### PNU maps from PRISMA data

**Figure 5:**
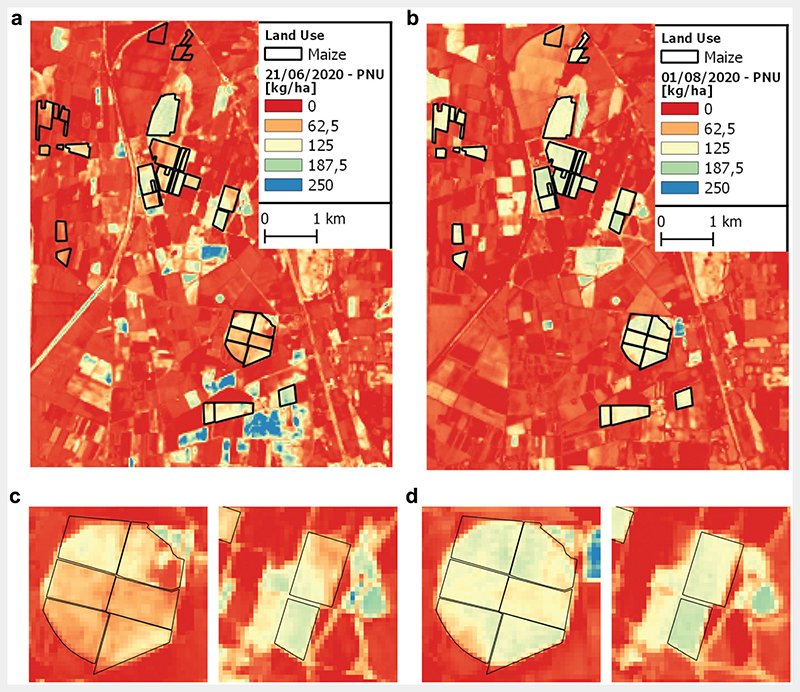
PNU maps and zoom on fields with different intra-fields sowing dates (c−d) on (a) 21 June 2020 and (b) 1 August 2020.

Figure 5 shows PNU maps, obtained from actual PRISMA data acquired on 21 June 2020 (Figure 5(a, c)) and 1 August 2020 (Figure 5(b,d)) with the proposed retrieval approach. The maps show a typical situation of summer Mediterranean cropland: a majority of land with low PNU values (reddish colours) and few fields with high PNU values (green-bluish colours). Low PNU values in the June image may represent either winter crop fields (in senescence phase or already harvested) or secondary crop fields with late sown (see southern portion of F1 in Figure 5 (c)) or alfalfa fields after mowing. High PNU values in June, besides the maize fields, correspond to sunflower and chickpeas, whose patches disappear in the August image as a consequence of harvesting. In August, the only fields in vegetative phase are represented by maize crop.

**Figure 6:**
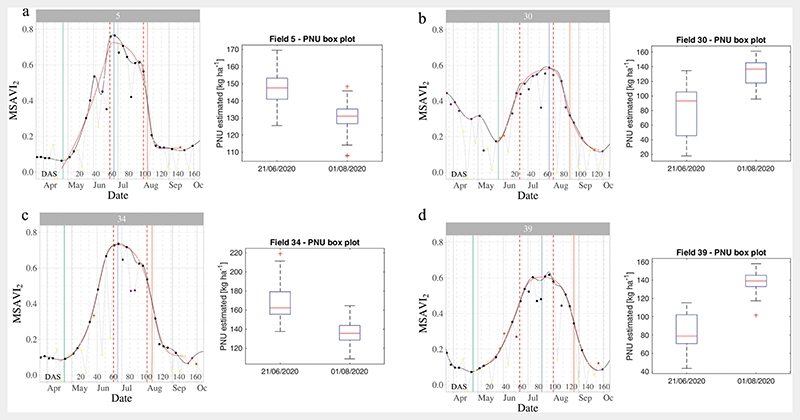
Temporal MSAVI and PNU boxplot in four different maize fields. Original and Savitzky−Golay smoothed Sentinel-2 data are reported as black dots and black line, respectively. The red line represents the double logistic curve used to compute the dates of start of season (green), peak of production (blue) and end of season (orange). The red dashed lines are the dates of PRISMA acquisitions for which PNU maps were calculated (21 June 2020 and 1 August 2020).

In order to evaluate the PNU maps generated from the proposed approach, boxplot diagrams of PNU values were produced for each maize field. These box-plots were compared to the temporal MSAVI trend of the same field. Figure 6 reports this comparison for four different fields, presenting different dynamics according to sowing dates, cultivated varieties and agro-practices. MSAVI plots show original (black dots) and Savitzky−Golay smoothed (black line) Sentinel-2 data and the double logistic curve (red line) used to assess crop phenological stages. The MSAVI analyses of the selected fields highlight situation where maize was in vegetative phase (Figure 6(b, d), late sowing) or already in reproductive phase (Figure 6(a,c), early sowing) during PRISMA acquisitions.

## Discussion

### Hybrid workflow configuration to estimate nitrogen uptake

Accuracy and error metrics from the cross-validation analysis demonstrated how GPR resulted in the best-performing algorithm (four times out of five best ranks − see Tables 6 and 7) among all the tested ML × DR combinations, for both BVs. In particular, the GPR-PCA20 configuration provided the absolute best-performing metrics in cross-validation: *R*^2^ = 0.97 and 0.68 and RMSE 0.11 g m^−2^ and 1.23 g m^−2^ for CCC and CNC, respectively. These results are in agreement with other studies found in the literature, confirming GPR algorithm to be a useful tool for the operational retrieval of biophysical variables from either multi-spectral (Upreti et al., 2019; Zhou et al., 2020) or hyperspectral (Berger, Verrelst, Féret, Hank et al., 2020; Candiani et al., 2022; Tagliabue et al., 2022; Verrelst et al., 2021) data. Another high-performing ML algorithm was NN, with 20 and 15 PCA components for CCC and CNC, respectively. Indeed, NN is used in different hybrid approach (Delloye et al., 2018), and it was selected as the base for operational products generated with Sentinel-2 data in the official ESA biopar processor SNAP (ATBD, Weiss, Baret et al., 2020). Recently, Upreti et al. (2019) tested different ML algorithms for the estimation of LAI, LCC, CCC and fractional cover, in two test sites located in Maccarese (Italy) and Shunyi (China). Regarding CCC, PLSR and RF resulted the best configurations for Maccharese and Shunyi, respectively. In another study, Liu et al. (2021) used RF in a hybrid retrieval schema for CCC but selecting it ***a priori*** with no comparison with other algorithm.

In conclusion, hybrid approaches are proposed as efficient inversion schemes, but no common agreement still exists about the best ML to be adopted. From the results achieved in this study, GPR and PCA transformation represented the best solution for CCC and CNC retrieval. This is in accordance with other papers which demonstrated that GPR with applied PCA ensures good estimation accuracy for crop BVs (Candiani et al., 2022; Danner et al., 2021; De Grave et al., 2020; Rivera-Caicedo et al., 2017; Tagliabue et al., 2022). Besides the good experimental results obtained in this study, performance and efficiency provided by coupling GPR and PCA are also confirmed by their intrinsic characteristic. GPR, being a kernel-based regression method, works well with small dataset and can provide uncertainty errors, in other words, an insight of how the algorithm is performing. Moreover, a spectral reduction based on the PCA approach has the benefit to exploit the full spectral signature and, at the same time, to reduce collinearity and redundancy typical of high-dimensional data like hyperspectral images.

#### BV retrieval performance and PNU relation

Performances of the best-identified retrieval configurations were validated with independent ground data, collected in real farm conditions, by sampling spatial heterogeneity of two fields with different sowing dates. The obtained results were more than satisfying as demonstrated by accuracy and error metrics: *R*^2^ of 0.79 and 0.84 and nRMSE of 19% and 15% for CCC and CNC, respectively. Scatterplots in Figure 3 show that data are well aligned on 1:1-line despite some overestimation for CCC and a slightly underestimation for CNC values greater than 6 g m^−2^ (60 kg ha^−1^). Validation results of CCC retrieval are comparable or even better than those reported in the literature, considering both hybrid approach using S2 data for wheat monitoring (*R*^2^ = 0.61 and nRMSE = 26%; Delloye et al., 2018; *R*^2^ = 0.74 and nRMSE = 23%; Upreti et al., 2019) and hyperspectral data from field spectroradio-metric measurements on heterogeneous grassland (*R*^2^ = 0.74 and nRMSE = 33%; Darvishzadeh et al., 2008).

For what concerns CNC, the results are very promising achieving a comparable performance when compared with traditional empirical approaches on multispectral data (RedEdge vegetation indices for maize: *R*^2^ = 0.79−0.87; Schlemmera et al., 2013) or more sophisticated combination of ML (PLSR for leaf nitrogen estimation) and RTM (for LAI) exploiting hyperspectral data (*R*^2^ = 0.85; S. Wang et al., 2021). Moreover, it is important to note that, with respect to other reported researches on maize (Crema et al., 2020; S. S. Wang et al., 2021), this study was performed on real field condition with no artificial nitrogen variability generated by different fertilisation levels. Therefore, the range of values is more limited and mainly related to natural spatial diversity within a typical cultivated field in the Mediterranean area. Other studies tested the hybrid approach exploiting the PROSAIL-PRO model. Berger et al. (2020) demonstrated retrieval feasibility using multitemporal field spectroradiometric measurements conducted along the entire crop season. The results showed a good correlation with field data, even though estimates from the developed hybrid nitrogen model resulted in more correlation with leaf and stalk nitrogen (i.e. PNU; *R*^2^ = 0.84−0.86 and RMSE = 2.32−2.15 g m^−2^) than with only leaf nitrogen (i.e. CNC; *R*^2^ = 0.69−0.71 and RMSE = 5.8−5.5 g m^−2^), which was strongly overestimated for medium−high nitrogen values. It is important to remark that, in Berger et al. (2020) study, field CNC was estimated in a different way: organ-specific N% (i.e. plant part) was multiplied by the corresponding dry mass per unit of ground area, whereas, in this study, CNC estimations were obtained from LNC, determined from leaf disk measurements with fixed area, multiplied by corresponding measured LAI. Moreover, our field dataset was limited to the vegetative phase (V2/3 and V11/12; Ritchie et al., 1989) when neither senescence nor cobs, hence translocation processes, were present. The same approach was recently adopted by Verrelst et al. (2021) to produce the first landscape canopy nitrogen maps from PRISMA. In that study, a GPR and active learning techniques (Verrelst, Dethier et al., 2016) were applied to the same dataset of Berger et al. (2020) in order to define a predictive CNC model to be applied to real spaceborne data. After active learning training (and including non-vegetated spectra), hybrid CNC model shows good performances (*R*^2^ = 0.69, RMSE = 3.42 g m^−2^ and nRMSE = 17%) but slightly worse than those obtained in our study (*R*^2^ = 0.84 and nRMSE = 15%). Finally, similar results to this study were obtained in Candiani et al. (2022) and Tagliabue et al. (2022), which share the same field protocol for CNC measurements, even though the latter adopted an active learning technique exploiting the available data to train the model.

Since PNU is the biophysical parameter of interest, used to perform “plant-based diagnosis” of crop N nutritional status using the concept of “critical N dilution curve” (Lemaire, 2021), CCC and CNC estimations were then compared to this variable to find the best BV-PNU model. CCC was strongly correlated with PNU (*R*^2^ = 0.80). This can also be explained considering that only a specific crop (maize) was investigated in limited vegetative phase. The result confirms the validity of the approach proposed in the literature for wheat (Baret et al., 2007; Delloye et al., 2018; Guerif et al., 2007) and maize (Crema et al., 2020; Schlemmera et al., 2013). However, the comparison with reported examples is not straightforward: the studies show different CCC−PNU performances and regression parameters and confounding terminology on which part of plant nitrogen uptake is considered (e.g. entire plant, only leaves/canopy, etc.). These aspects highlight the empirical nature of CCC−PNU relation. This is also reported in other studies that underline how the chlorophyll-based relation with nitrogen is moderately strong across different species and dependent on crop status (Berger, Verrelst, Féret, Hank et al., 2020; Berger, Verrelst, Féret, Wang et al., 2020; Homolová et al., 2013). Under this perspective, PNU estimated from CCC may not be transferable in different contexts.

On the other hand, the identified CNC−PNU relation, besides slightly better (*R*^2^ = 0.82), appears to be physiologically based, being leaf-level nitrogen uptake a part of the entire plant nitrogen sink. As a matter of fact, plant nitrogen is strictly bound to proteins, while chlorophyll pigments represent only a part of it as reported in the work by Kokaly et al. (2009) and Berger et al. (2020). In our study, estimated CNC resulted, on average, about 30% of PNU in different analysed conditions (e.g. F1 and F2) which is compatible to what reported in the literature for maize hybrids (Bender et al., 2013; Berger, Verrelst, Féret, Hank et al., 2020). In this context, traditional agronomic experiment (available or to be specifically conducted) could be used to develop allometric relations between organ-specific (e.g. canopy level) and total plant N uptake. Assimilation of CNC in crop models can be considered a powerful alternative to estimate nitrogen partitioning in the plant (Weiss, Jacob et al., 2020). These considerations candidate CNC as a more direct way to estimate PNU from spectral data without using empirical relation with another variable, such as chlorophyll. From an operational point of view, PNU estimated from CNC should be more robust and transferable across seasons and crops.

#### Nitrogen uptake mapping and implication for precision agriculture and crop monitoring

The best CNC hybrid model (GPR algorithm with PCA-20) was applied to 2020 PRISMA acquisitions for the generation of CNC maps on the study area. The relation between CNC and PNU was then used to map the spatial distribution of PNU in all the maize fields identified from the official common agricultural policy declarations (Figure 5). PNU maps for maize fields showed values within expected ranges, except for the peak, when PNU reaches values of about 180−200 kg ha^−1^ (Bender et al., 2013; Berger, Verrelst, Féret, Hank et al., 2020).

Intra-field variability from PNU maps provides useful information related to crop development in early pheno-logical stages. This is more evident from the first PRISMA image acquired in late June, in which some maize fields with late sowing date show plants not yet in full development (Figure 5(c)). It is also interesting to discuss the temporal evolution of PNU estimation in relation to crop conditions and phenological development. Crop dynamics for each investigated field were reconstructed using the time-series analysis provided by “sen2rts” R package (L. Ranghetti et al., 2021). Analysis of MSAVI trend of each field, derived from S2 imagery, allowed estimating dates of important crop phases such as “start of season”, “peak of production” and “end of season”. PNU estimation for the two dates resulted in agreement with the expected nitrogen uptake trend from the plant, showing an increase before the reproductive stage up to the “peak of production” and a reduction afterwards due to senescence and translocation to reproductive organs (see boxplot diagrams in Figure 6).

PNU maps produced from spaceborne hyperspectral data (e.g. PRISMA) can be exploited for several applications; in this context, user needs would determine what would be the most useful maps to consider. In case of precision farming workflows devoted to provide information to end-user for agro-management, an active interaction with farmers is expected. Based on sowing dates, farmers will exploit geo-products provided at time suitable to support fertilisation. Indeed, mapping the plant nitrogen spatial variability information within fields during leaf development stages (V3 − V13, Ritchie et al., 1989) is crucial for the generation of prescription maps; these data can be directly exploited by variable rate technology machinery for the application of site- -specific fertilisation (Crema et al., 2020).

In case of system devoted to monitor district (regional) crop condition or to estimate yield and grain quality (Busetto et al., 2017), the most usable proxies for final yield estimation are remote-sensing products related to plant nitrogen acquired close to the peak of season (about flowering; Wang et al., 2021). Crema et al. (2020) demonstrated that nitrogen nutritional index maps derived by remote-sensing products can be a good proxy to identify statistical difference in maize production among fields. This study showed that, for an operational nitrogen monitoring system, PNU information obtained from EO data at field level needs to be integrated by Sentinel-2 decametric data to identify specific crop stages.

### Conclusions

In this study, an operational workflow for the mapping of the plant nitrogen uptake from space imaging spectroscopy was developed. The work proposed a hybrid method, which combines the radiative transfer model PROSAIL-PRO and different machine learning regression algorithms (GPR, NN, PLSR, SVR and RF), for the estimation of CCC and CNC as a future proxy for PNU mapping. The use of a hybrid method ensures the exportability of the procedures and a fast processing. Dimensionality reduction methods (PCA) were used to condense the spectral data into components and to analyse their influence on the models. GPR algorithms trained with PCA-20 resulted as the most suitable models for both CCC and CNC. The independent validation against ground data showed good performances: *R^2^* = 0.79, nRMSE = 19% for CCC and *R^2^* = 0.84, nRMSE = 15% for CNC. These models were subsequently applied to PRISMA-like images, acquired in 2018 over the study area, and the relationship between estimated values of CCC and CNC with measured values of PNU was investigated. PNU was slightly more correlated to CNC (*R*^2^ = 0.82) than CCC (*R*^2^ = 0.80). Therefore, the hybrid model of CNC and the CNC−PNU relation was applied to real PRISMA images, acquired in 2020 over the study area, for the generation of PNU maps.

Result obtained from this study show how crop traits, in particular maize traits, can be estimated from space using data from new-generation hyper-spectral sensors. The hybrid approach, which is fully independent from field measurements and datasets, led to good performance and accuracy during the validation phase against the test site data. Moreover, PNU was estimated within the expected ranges, and its dynamics resulted in agreement with the usual plant nitrogen uptake.

## Figures and Tables

**Table 1:** Crop traits at leaf (L), canopy (C) and plant (P) level.

Level	Parameter	Unit	ESU	Range	Method
L	LCC	μg cm^−2^	87	25.2−54.4	Indirect
L	LMA	g cm^−2^	31	0.0040−0.0054	Destructive
L	LNC	mg cm^−2^	31	0.072−0.197	Derived
C	LAI	_m_2 _m_-2	87	0.11−5.70	Indirect
C	CCC	g m^−2^	87	0.05−2.88	Derived
C	CNC	g m^−2^	31	0.22−9.44	Derived
P	Biomass	kg m^−2^	27	0.18−10.00	Destructive
P	PNC	%	27	0.9−4.1	Destructive
P	PNU	kg ha^−1^	27	70−162	Derived

LCC: leaf chlorophyll content; LNC: leaf nitrogen content; LAI: leaf area index; PNC: plant nitrogen content; CCC: canopy chlorophyll content; CNC: canopy nitrogen content; PNU: plant nitrogen uptake.

**Table 2:** Details of EO datasets used in this study: year of the dataset, sensor, number of available images, bands, ground sampling distance and acquisition dates (original values are reported in round brackets).

Year	Sensor	Images	Bands	GSD	Dates
2018	PRISMA-like (HyPlant-DUAL)	2	150 (480)	1 m 4.5 m	7 July 2018 30 July 2018
2020	PRISMA	2	150 (231)	30 m	21 June 2020 01 August 2020
2020	Sentinel-2	31	10 (12)	10 m	From March to November

**Table 3:** Advantages and limitations/critical aspects of hybrid approach for crop traits retrieval.

	Advantages	Limitations and critical aspects
RTM	Use of RTM allows to generate a wide range of physically sound crop spectra Besides sun-target-sensor geometry and background, database generation needs only crop trait ranges and their distributions as input Flexible generation of database cardinality	In some real cases, RTM assumptions may not be valid Simulated spectra rely on proper calibration of selected RTM Potential un-realistic combination of crop traits in simulation
MLRA	• MLRA is a flexible and computational-efficient method to solve not linear relationships between spectra and crop traits	MLRA sometimes can be considered a sort of “black box” Limited training database and/or incorrect tuning setup may lead to overfitting or underfitting solutions
Hybrid	Field data are not required in the hybrid training phase RTM+MLRA provides more robust models across space (different agro-sites) and time (different crop seasons)	Hybrid model can only retrieve crop traits in the training database ranges Possible computational-demanding solutions Require validation with independent dataset to assess transferability

**Table 4:** Parametrization of the leaf (PROSPECT-PRO) and canopy (4SAIL) model with unit of measurements, ranges and distributions coming from the field campaign.

	Param.	Description	Unit	PDF	Range^a^	
PROSPECT-PRO	N	Structural parameter	-	Normal	1.4	0.14
	LCC	Chlorophyll content	μg cm^−2^	Normal	41.5	8.8
	Ccx	Carotenoid content	μg cm^−2^	Normal	7.32	1.5
	Canth	Anthocyanin content	μg cm^−2^	Normal	0	0
	Cbp	Brown pigment	_μg cm_^−2^	Normal	0	0
	Cw	Water content	mg cm^−2^	Normal	12.92	1.91
	Cp	Protein content	g cm^−2^	Uniform	0	0.001
	CBC	Carbon-based constituents	g cm^−2^	Uniform	0.003	0.006
4SAIL	ALA	Average leaf angle	d^e^g _2_	Normal	49	4.9
	LAI	Leaf area index	m2 _m_-2	Normal	1.77	1.4
	HOT	Hot spot parameter	m m	Normal	0.01	0.001
	SZA	Solar Zenith Angle	deg	Uniform	26	30
	OZA	Observer Zenith Angle	deg	Uniform	0	0
	RAA	Relative Azimuth Angle	deg	Uniform	0	0
	BG	Soil spectra	-	Uniform	1	4

a min and max values in case of uniform PDF; *μ* and *σ* values in case of normal PDF.

**Table 5:** Brief description and references of the algorithms tested in this study.

Algorithm	Brief Description	References
PLSR	A technique that reduces the predictors to a smaller set of uncorrelated components and performs least squares regression on these components, instead of on the original data.	(Geladi & Kowalski, 1986)
GPR	A kernel-based regression method that uses a stochastic probability distribution-based process for providing estimates with their level of uncertainties.	(Rasmussen & Williams, 2006)
SVR	A technique for investigating the relationship between a small set of training data samples (called support vector) and a real-valued variable.	(Zhang & O’Donnell, 2020)
NN	Composed of many layers of artificial neurons that transform input data into outputs while learning how to minimize the chance for errors and unwanted results.	(Schmidhuber, 2015)
RF	It operates by constructing several decision trees during training time and outputting the mean of the classes as the prediction of all the trees.	(Breiman, 1996, Breiman, 2001)

**Table 6:** Cross-validation metrics of hybrid MLRAs and DR tested for CCC retrieval: Mean Absolute Error (MAE, g m^−2^), Root Mean Squared Error (RMSE, g m^−2^), relative RMSE (rRMSE, %), normalised RMSE (nRMSE, %) and coefficient of determination (*R*^2^).

MLRA	DR	MAE	RMSE	rRMSE	nRMSE	*R^2^*
GPR	PCA-20	0.053	0.114	16.684	2.562	0.971
GPR	PCA-15	0.055	0.119	17.394	2.671	0.969
GPR	PCA-10	0.058	0.126	18.484	2.838	0.964
NN	PCA-20	0.064	0.119	17.501	2.687	0.968
GPR	PCA-5	0.067	0.150	21.911	3.364	0.950
NN	PCA-15	0.068	0.123	17.992	2.763	0.966
NN	PCA-10	0.072	0.132	19.325	2.967	0.961
NN	PCA-5	0.080	0.155	22.717	3.488	0.946
RF	PCA-10	0.082	0.166	24.357	3.740	0.939
RF	PCA-5	0.082	0.170	24.894	3.823	0.936
RF	PCA-15	0.082	0.166	24.302	3.732	0.939
RF	PCA-20	0.083	0.166	24.283	3.729	0.939
SVR	PCA-20	0.099	0.184	27.017	4.148	0.931
SVR	PCA-15	0.101	0.188	27.588	4.236	0.928
SVR	PCA-10	0.104	0.201	29.434	4.520	0.920
PLSR	PCA-5	0.107	0.166	24.333	3.736	0.938
PLSR	PCA-10	0.111	0.164	23.954	3.678	0.940
PLSR	PCA-20	0.111	0.164	24.050	3.693	0.940
PLSR	PCA-15	0.111	0.164	24.045	3.692	0.940
SVR	PCA-5	0.114	0.222	32.582	5.003	0.902

Results ordered according to MAE values.

**Table 7:** Cross-validation metrics of hybrid MLRAs and DR tested for CNC retrieval: Mean Absolute Error (MAE, g m^−2^), Root Mean Squared Error (RMSE, g m^−2^), relative RMSE (rRMSE, %), normalised RMSE (nRMSE, %) and coefficient of determination (*R*^2^).

MLRA	DR	MAE	RMSE	rRMSE	nRMSE	*R^2^*
GPR	PCA-20	0.804	1.236	67.294	7.454	0.680
GPR	PCA-15	0.806	1.238	67.380	7.464	0.679
GPR	PCA-10	0.827	1.283	69.832	7.735	0.656
NN	PCA-15	0.835	1.243	67.679	7.497	0.677
GPR	PCA-5	0.846	1.299	70.738	7.836	0.647
NN	PCA-20	0.849	1.272	69.246	7.670	0.662
NN	PCA-10	0.853	1.311	71.350	7.903	0.641
NN	PCA-5	0.855	1.294	70.429	7.801	0.650
RF	PCA-15	0.856	1.333	72.578	8.039	0.629
RF	PCA-20	0.858	1.335	72.699	8.053	0.628
RF	PCA-10	0.872	1.377	74.960	8.303	0.603
SVR	PCA-20	0.876	1.375	74.882	8.295	0.607
SVR	PCA-15	0.879	1.388	75.579	8.372	0.601
RF	PCA-5	0.881	1.385	75.404	8.352	0.599
PLSR	PCA-5	0.893	1.314	71.548	7.925	0.639
SVR	PCA-10	0.895	1.438	78.303	8.673	0.571
SVR	PCA-5	0.902	1.456	79.287	8.782	0.562
PLSR	PCA-10	0.905	1.274	69.358	7.683	0.661
PLSR	PCA-15	0.912	1.278	69.581	7.707	0.659
PLSR	PCA-20	0.912	1.278	69.584	7.708	0.659

Results ordered according to MAE values.
